# Dynamic Evolution of the Glymphatic System at the Early Stages of Subarachnoid Hemorrhage

**DOI:** 10.3389/fneur.2022.924080

**Published:** 2022-07-01

**Authors:** Changkai Hou, Jian Li, Bangyue Wang, Quanlei Liu, Yan Zhao, Hao Zhang, Weihan Wang, Wen Ren, Xiaopeng Cui, Xinyu Yang

**Affiliations:** ^1^Department of Neurosurgery, Tianjin Medical University General Hospital, Tianjin, China; ^2^Department of Neurosurgery, Nanjing Drum Tower Hospital, The Affiliated Hospital of Nanjing University Medical School, Nanjing, China; ^3^Department of Neurosurgery, Beijing Friendship Hospital, Capital Medical University, Beijing, China; ^4^Department of Radiology, The Affiliated Suzhou Hospital of Nanjing Medical University, Suzhou, China; ^5^Department of Neurosurgery, Tianjin Fifth Central Hospital, Tianjin, China

**Keywords:** glymphatic system, subarachnoid hemorrhage, dynamic evolution, AQP4, dystrophin-associated complex

## Abstract

The early stages of subarachnoid hemorrhage (SAH) are extremely important for the progression and prognosis of this disease. The glymphatic system (GS) has positive implications for the nervous system due to its ability to clearance tau and amyloid-β (Aβ) protein. Previous studies have shown that GS dysfunction will appear after SAH. However, there is no systematic evaluation of the degree of damage and development process of GS function in the early stage after SAH. In this study, we evaluated the GS function and neurobehavioral in the sham, 6 h, 1, 3, and 7 days after SAH, respectively. Our results showed that the function of GS was severely attenuated in mice after SAH with a decreased polarity of Aquaporin-4 (AQP4), increased expression of AQP4, a linear correlation with the dystrophin-associated complex (DAC), the proliferation of reactive astrocytes, increased tau protein accumulation, and decreased neurological function. Collectively, these findings provide a comprehensive understanding of the functional changes of GS after SAH, provide references for subsequent scholars studying SAH, and suggest some potential mechanistic insight that affects AQP4 polarity and GS function.

## Introduction

Subarachnoid hemorrhage (SAH) accounts for 2–7% of all stroke disorders but accounts for a large proportion of the mortality and disability from stroke due to its younger age of onset and high rates of both mortality and disability (1). The early stage after SAH has an important influence on the development progression and prognosis of the disease, especially in the first 3 days after SAH. Scholars have generally referred to it as early brain injury (EBI), and the complex physiopathological mechanisms of EBI have caused an increasing number of studies to focus currently on the early stage of SAH (2–4).

The glymphatic system (GS), a new concept proposed in recent years, has received extensive attention due to its clearance of interstitial solutes and its important role in intracranial diseases (5, 6). Currently, available data suggest that the functional impairment of GS after SAH is not only present in rodents but also in non-human primates (7–9).

The damage of the GS appeared immediately after SAH, and the function of GS was still impaired 1 week after SAH, which continued to affect the occurrence and development of neuroinflammation and neuronal apoptosis (7, 9). This suggests that targeting the improvement of GS potentially serves as a new strategy for the treatment of SAH. At present, some scholars have used different drugs to conduct preliminary research and achieved relatively positive curative effects (10, 11). However, the dynamic evolution of early GS functional changes after SAH and the systematic study of aquaporin 4 (AQP4) expression and polarization have been lacking.

## Materials and Methods

### Animals

All experiments described in this study were approved by the Tianjin Medical University General Hospital Animal Care and Use Committee. All the mice used were 8–12-week-old C57BL/6 male mice, purchased from Vital River Laboratory Animal Technology Co., Ltd. (Beijing, China).

### SAH Model

The SAH model was created by transfusing blood into the cisterna magna as previously described (12). Briefly, mice were anesthetized with a mixture of ketamine (100 mg/kg) and xylazine (10 mg/kg) by intraperitoneal injection (ip), and then 60 μl of fresh non-heparinized blood was drawn from the apex of the heart. After that, the mice were immobilized on a stereotaxic instrument, the cisterna was exposed, and the syringe pump was carefully inserted ~1 mm under the atlantooccipital membrane. The microsyringe was kept in place for 3 min after injection of blood (30 μl/min for a total of 60 μl) to avoid leakage of blood or CSF. After suturing, mice were placed on a constant temperature heating pad 30° below the head for better diffusion of blood to the subarachnoid space until awakening from anesthesia.

### Intracisternal Tracer Infusions

Mice were anesthetized and immobilized in a stereotactic frame. Cerebrospinal fluid (CSF) tracer was rhodamine B isothiocyanate dextran (Sigma; catalog no. R9379, MW70KDa) formulated at a concentration of 0.5% in artificial CSF. CSF tracer was injected into the cisterna magna *via* a 33 GA syringe pump at 2 μl/min for 5 min (10 μl total volume). After 30 min of the start of the infusion, anesthetized animals were transcardially perfused. Brains were removed and post-fixed overnight in 4% PFA and sliced into 100 μm coronal sections. The movement of CSF tracers along perivascular spaces and penetration into the brain parenchyma were visualized by conventional fluorescence microscopy (13). To minimize environmental and human errors as much as possible, slices from all different groups were recorded rapidly under the same fluorescence conditions on the same day by a blinded investigator, and the whole brain fluorescence intensity and whole cortical fluorescence intensity was evaluated using Fiji software.

### Immunofluorescence

Under deep anesthesia, the brains were removed for fixation with 4% paraformaldehyde overnight after transcardial perfusion of the mice, followed by gradient dehydration. Using a cryostat to cut 30 μm thick slices, according to experimental experience, this thickness of GFAP/AQP4 leads to better fluorescence without stripping. Primary antibodies were polyclonal rabbit anti-AQP4 (1:200, ab46182, Abcam) and polyclonal Goat anti GFAP (1:250, ab48004, Abcam). DAPI staining was performed after incubation with fluorescence conjugated secondary antibodies. Under confocal laser scanning microscope or fluorescence microscope, the observation sites of each brain slice included bilateral cortex, ventral cortex, and dorsal cortex. One field of view was randomly selected for all the observation sites. GFAP expression, AQP4 expression, and AQP4 polarization were assessed using Fiji (ImageJ) software.

### Evaluating AQP4 Polarization

Aquaporin-4 polarization was evaluated as previously described (13, 14), Briefly, AQP4 polarization is the relative measurement of AQP4 localization. Relative to parenchymal AQP4 immunoreactivity, increased polarity indicates higher perivascular AQP4 immunoreactivity and decreased polarity indicates lower perivascular AQP4 immunoreactivity. The percentage of the area in which AQP4 immunofluorescence was greater than or equal to perivascular AQP4 immunofluorescence was measured by thresholding analysis after we measured the median immunofluorescence intensity in the perivascular area is referred to as “AQP4% area.” AQP4 polarization was expressed as the percentage of the area where AQP4 immunoreactivity was lower than the perivascular end (“polarization” = 100–AQP4% area).

### Western Blot

Western blotting (WB) was performed as previously described (15). Proteins from brain samples were lysed using RIPA lysis buffer. The supernatant was mixed with loading buffer until a concentration of 5 μg/ml was reached. Protein samples (20 μg) were loaded onto SDS-PAGE gels (10%), followed by electrophoresis, and then transferred onto nitrocellulose membranes (0.45 μm). The membrane was incubated with 5% non-fat milk for 1 h at room temperature and then incubated with primary antibodies overnight at 4°C. The primary antibodies included the following: anti-β-actin (ab8227, Abcam), anti P-tau (pS396, catalog #44–752G, Life Technologies), anti-AQP4 (ab46182, Abcam), and anti-GFAP (ab48004, Abcam). Membranes were visualized for immunoblots using the ECL plus chemiluminescence kit after 1 h of processing with secondary antibodies and then quantified using Fiji (ImageJ) software.

### Real-Time PCR

Total RNA purification and cDNA synthesis were performed using Trizol reagent and YEASEN gDNA digester plus (YEASEN, China), respectively, following the manufacturer's protocols. qRT-PCR was performed using Bio-Rad's CFX connect Real-Time PCR System. Each cDNA sample was diluted five-fold and amplified in a 20-μl volume using the Hieff UNICON Universal Blue qPCR SYBR Green Master Mix (YEASEN, China) with 1,000 nM final concentrations of each primer. The amplification cycles consisted of an initial denaturing cycle at 95°C for 2 min, followed by 40 cycles of 10 s at 95°C and 30 s at 60°C. The RNA quantities were normalized to β-actin before calculating the relative expressions of AQP4, DAG1, DMD, SNTA1, and DTNA. Relative quantification of the target gene was analyzed using the 2-^Δ*ΔCt*^ method as described previously (16). The primer sequence for qPCR is presented in Table 1.

**Table 1:** Primer sequence for real-time PCR.

**Gene**	**Sequence**
AQP4	Forward primer: CTTTCTGGAAGGCAGTCTCAG Reverse primer: CCACACCGAGCAAAACAAAGAT
β-actin	Forward primer: AGGGAAATCGTGCGTGACAT Reverse primer: TCCAGGGAGGAAGAGGATGC
DAG1	Forward primer: GAGCAGTGAGGACGATGTTTA Reverse primer: CCCTTCCTCTTCTTGCGATAG
DMD	Forward primer: GCTTCTAGTCATCTGGGCTTATC Reverse primer: ACATCTGGAGCCCTAGACAATA
DTNA	Forward primer: GTCCACTTCACACCTCTTTAGTT Reverse primer: GAAAGGTCCTCAGGAAGAATGG
SNTA1	Forward primer: GATTGGCTGGCTGACAGAA Reverse primer: CTGAGGGAGAGAGCAGTAGAA

### Brain Water Content

Mice were anesthetized and the brain tissues from the sham group, 6 h, 1, 3, and 7 days after SAH, were removed. The wet weight was determined by instantly weighing the samples removed from the brainstem and cerebellum. The samples were then placed in an oven (100°C) for 3 days to obtain the dry weight. The brain water content was calculated as follows: [(wet weight–dry weight)/(wet weight)] × 100%.

### Cerebral Perfusion Analysis

A laser speckle imager (pericam PSI system, perimed AB, Sweden) was used to assess cerebral cortical perfusion throughout the convexity. The system was set to a laser irradiation area of 2 cm × 2 cm and a detection distance of 10 cm. The review software (PIMsoft software version 1.2) is used to evaluate perfusion data. The average of the fixed-size regions of interest was selected for cerebral cortical perfusion over the time-period of interest. In this part of the experiment to reduce error, the measurement of laser speckle was performed in a way that each mouse was continuously monitored. Mouse cerebral cortical perfusion values were first measured after unambiguous marking of healthy mice, after which the SAH model was made for each punctuated mouse, and the perfusion of each mouse at 5 min, 6 h, 1, 3, and 7 days after SAH was measured sequentially. For each mouse, the perfusion volume before modeling was taken as the baseline, and the perfusion volume at different time points after SAH was compared with the baseline, respectively, and the changes from baseline and at different time points after SAH were statistically analyzed.

### Intracranial Pressure

Intracranial pressure (ICP) was monitored in sham, 6 h, 1, 3, and 7 days, after SAH using the Transonic Scisense SP200 Data Acquisition system (Transonic, USA). The mice were anesthetized and fixed on the fixed frame, and a bone hole of 0.8 mm diameter at 2 mm posterior to the anterior fontanel and 2.5 mm lateral to the left was created. After calibration of the ICP monitoring device, a 1.6F piezoelectric flexible pressure probe (Transonic FTH-1211B-0012) was inserted 2 mm below the cerebral cortex through the bone hole. Real-time ICP data were observed using LabScribe. ICP was recorded at a sampling speed of 1.0 samples/s. The data were recorded and saved, and the average value within the observation period was used as the recorded value.

### Neurological Assessment

The 8-test sensorimotor neuroscore was used to evaluate neurobehavioral function. The score assessed the functional performance of exploration, climbing, forelimb and hind limb use, beard and lateral sensation, balance, and visual reflex. Scores ranged from 0 to 24, with higher scores indicating better function (17).

### Statistical Analysis

Data were presented as mean ± SEM. Statistical analyses were carried out using Graphpad Prism. One-way ANOVA followed by Tukey's multiple comparisons test was used to compare multiple groups. Linear regression analysis was used to analyze paired data. A *P* <0.05 was considered statistically significant.

## Results

### GS Dysfunction Appears in the Early Stages After SAH

**Figure 1:**
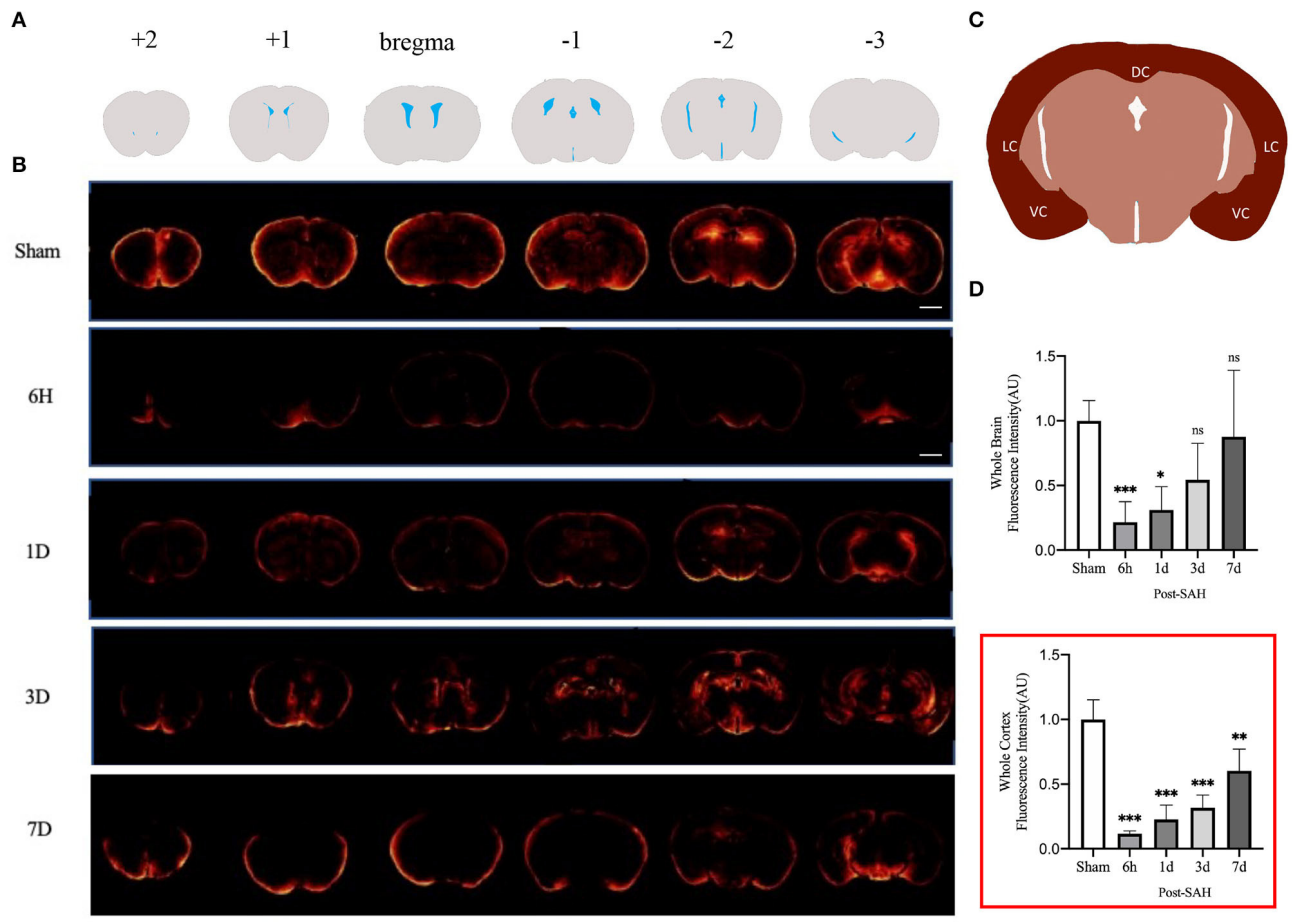
GS CSF tracer penetration images and statistical results. **(A)** Schematic representation of different layers of fluorescent tracers; **(B)** Representative fluorescence schematics of different groups, Scale bar, 2 mm; **(C)** Schematic of cortex; DC, dorsal cortex; VC, ventral cortex; LC, lateral cortex; **(D)** Whole-brain CSF tracer penetration intensity and whole-brain cortex CSF tracer penetration intensity (red box) analysis statistical diagram; after SAH, the CSF tracer penetration intensity of each group was significantly lower than that of the sham group and exhibited a gradually recovered state; *n* = 4–5/group, *P < 0.05, **P < 0.01, ***P < 0.001; ns, not statistically significant. One-way ANOVA, Tukey's *post-hoc* test.

We assessed GS function in sham, 6 h, 1, 3, and 7 days, mice using cisterna magna injection of a fluorescent CSF tracer. As shown in Figures 1A,B, compared with the sham group, all the mice in the SAH group had GS dysfunction. For a more accurate analysis of GS function, we analyzed the whole-slice CSF tracer penetration and cortical CSF tracer penetration intensity of six different anatomical layers, including bregma +2 mm, bregma +1 mm, bregma, bregma −1 mm, bregma −2 mm, and bregma −3 mm, as shown in the Supplementary Material. We totaled the whole-section CSF tracer penetration intensity of six anatomical layers in each mouse to represent the whole-brain CSF tracer penetration intensity and normalized it. As shown in Figure 1D, the whole-brain CSF tracer penetration intensity was significantly attenuated in the 6 h and 1 day groups after SAH compared with the sham group (*P* = 0.0065, *P* = 0.0253), but there was no significant difference between the 3 and 7 days groups after SAH compared with the sham group (*P* = 0.2068, *P* = 0.9711).

Current GS research is more likely to evaluate the cortex. We further analyzed the CSF tracer penetration intensity of the GS in different anatomic layers in mice, and the selected cortical schematic is shown in Figure 1C. We totaled the cortical CSF tracer penetration intensity of six anatomical layers in each mouse to represent the whole cortical CSF tracer penetration intensity and normalized it. As shown in the red box in Figure 1D, the whole cortical CSF tracer penetration intensity was significantly attenuated in the 6 h, 1, 3, and 7 days groups after SAH compared with the sham group (*P* < 0.0001, *P* < 0.0001, *P* < 0.0001, *P* = 0.0017). Further intergroup comparisons were performed for the four groups after SAH. The CSF tracer penetration intensity of the 6 h group after SAH was the lowest, and the GS function was the worst. Subsequently, the GS function of each group gradually recovered. However, no significant difference was observed between the 6 h group and the 3 days group (*P* = 0.1301), or between the 1 day group and the 3 days group (*P* = 0.8189), but a significant difference was observed between the 3 days group and the 7 days group (*P* = 0.0242), indicating that the recovery of GS function was not obvious within 3 days after SAH, but entered a faster recovery process from 3 to 7 days after SAH.

**Figure 2:**
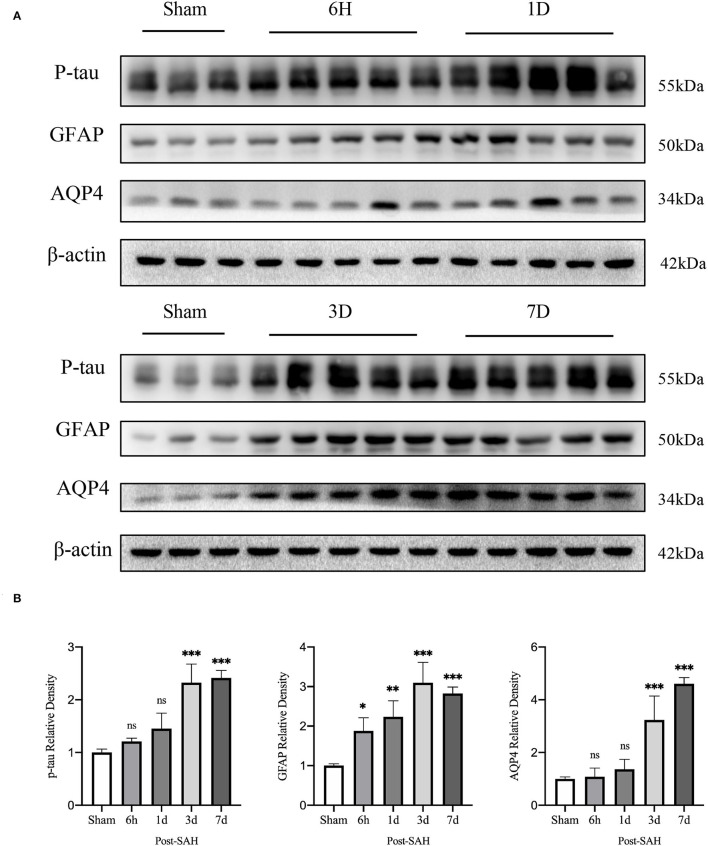
Results of western blot analysis of AQP4, GFAP, and p-tau in different groups. **(A)** Representative bands by WB. **(B)** Venus quantification of WB bands for AQP4, GFAP, and p-tau in brain homogenates from various groups of mice after normalization with internal reference genes. Compared with the sham group, the expression of p-tau showed no significant increase at 6 h and 1 day, but significant increase at 3 and 7 days (*P* = 0.7177, *P* = 0.0908, *P* < 0.0001, *P* < 0.0001). Compared with the sham group, GFAP expression was significantly increased at 6 h, 1, 3 and 7 days (*P* = 0.0228, *P* = 0.0013, *P* < 0.0001, *P* < 0.0001). Compared with the sham group, the expression of AQP4 showed no significant increase at 6 h and 1 days, but a significant increase at 3 and 7 days (*P* = 0.9994, *P* = 0.8447, *P* < 0.0001, *P* < 0.0001). *P < 0.05, **P < 0.01,***P < 0.001; ns, not statistically significant. One-way ANOVA, Tukey's *post-hoc* test.

Interstitial metabolic wastes, such as tau protein can be excreted by the GS. Since GS function was severely disrupted after SAH, we wondered whether this change was accompanied by the accumulation of parenchymal tau protein deposits. Our experiment evaluated the amount of P-tau protein expression using western blotting. The representative bands are shown in Figure 2A, and the statistical results are shown in Figure 2B. No significant difference was observed between the 6-h group and the 1-day group compared with the sham group (*P* = 0.7177, *P* = 0.0908), whereas P-tau protein expression was significantly redundant between the 3-days group and the 7-days group compared with the sham group (*P* < 0.0001, *P* < 0.0001). Further analysis showed that the accumulation of P-tau protein in SAH gradually increased and peaked at 3 days, and there was no sign of reduction at 7 days. This finding confirms the importance of the GS for P-tau protein transport while also illustrating that, in pathological situations, as GS function is compromised, interstitial metabolic waste gradually accumulates and cannot be alleviated by self-compensation within a short period of time.

### Cortical AQP4 Expression Changes Are Location- and Time-Dependent

As indicated in the methods, the immunofluorescence locations of each slice were the dorsal cortex, ventral cortex, and the lateral cortex. The expression of AQP4 and GFAP and the polarity of AQP4 were calculated at different locations.

**Figure 3:**
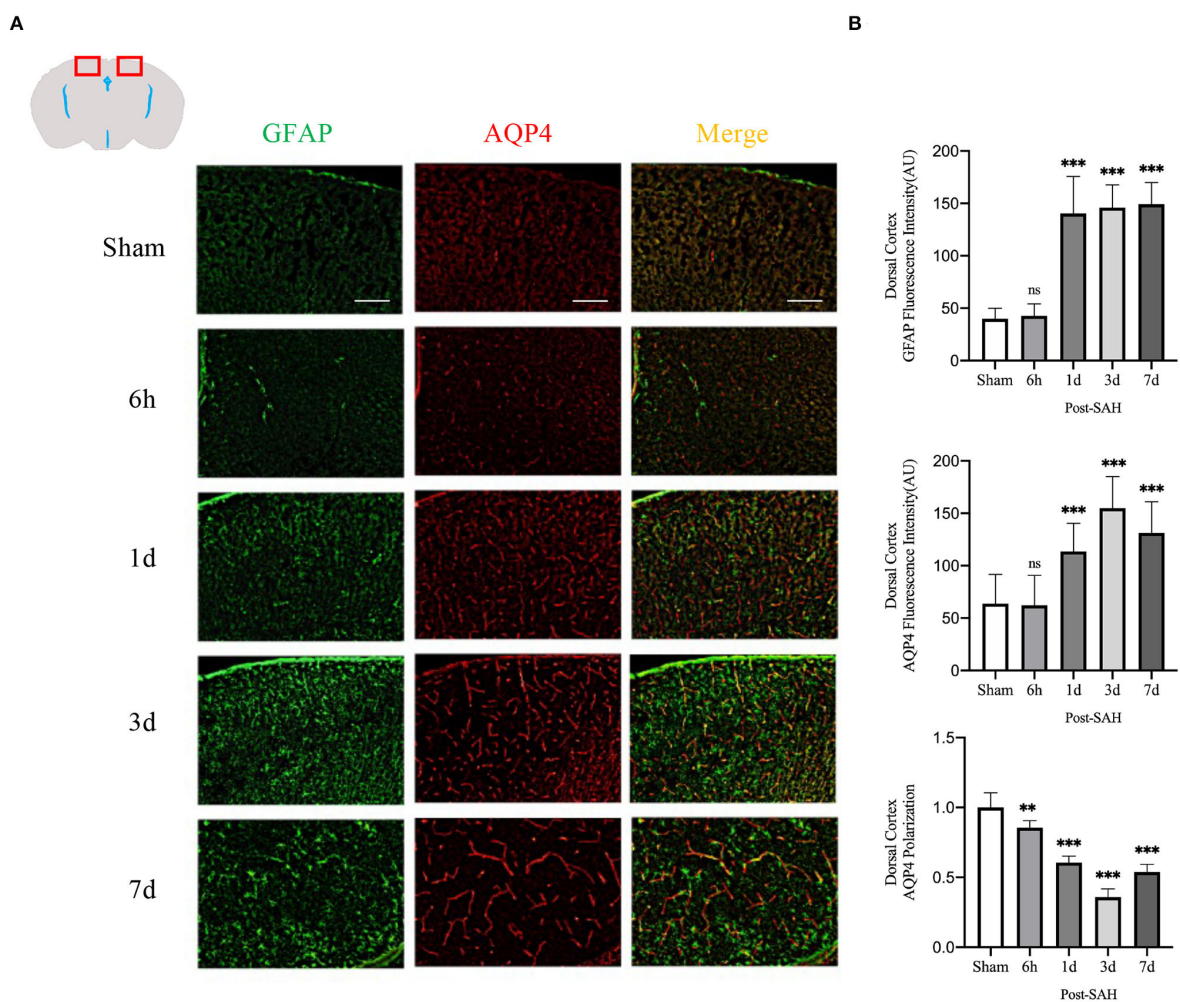
AQP4 and GFAP fluorescence maps among different groups in the dorsal cortex. **(A)** Schematic diagram of the dorsal cortex along with representative immunofluorescence maps, Scale bar, 100 μm; **(B)** Statistical plots of AQP4 mean fluorescence intensity, GFAP mean fluorescence intensity, and polarity of AQP4 in the dorsal cortex of different groups of mice. Compared with the sham group, the mean fluorescence intensity of AQP4 did not change significantly at 6 h but increased significantly at 1, 3, and 7 days (*P* = 0.9999, *P* = 0.0007, *P* < 0.0001, *P* < 0.0001; *n* = 8–24/group). Compared with the sham group, the mean fluorescence intensity of GFAP did not change significantly at 6 h but increased significantly at 1, 3 and 7 days (*P* = 0.9942, *P* < 0.0001, *P* < 0.0001, *P* < 0.0001; *n* = 8–24/group). The polarity of AQP4 decreased significantly at 6 h, 1, 3, and 7 days compared with the sham group (*P* = 0.0086, *P* < 0.0001, *P* < 0.0001, *P* < 0.0001; *n* = 5–6/group). **P < 0.01, ***P < 0.001; ns, not statistically significant. One-way ANOVA, Tukey's *post-hoc* test.

The mean CSF tracer penetration intensity of AQP4 and GFAP and the polarity of AQP4 were calculated for the dorsal cortex, as shown in Figures 3A,B. Analysis of the mean fluorescence intensity of AQP4 showed that there were no significant differences between the 6-h group and the sham group (*P* = 0.9999), but that at 1, 3, and 7 days, it was significantly higher than that of the sham group (*P* = 0.0007, *P* < 0.0001, *P* < 0.0001). Analysis of the mean fluorescence intensity of GFAP showed that the mean CSF tracer penetration intensity at 6 h after SAH was not significantly different from that of the sham group (*P* = 0.9942) and that at 1, 3, and 7 days, it was significantly higher than that of the sham group (*P* < 0.0001, *P* < 0.0001, *P* < 0.0001). Analysis of the polarity of AQP4 revealed that the polarity of AQP4 was significantly decreased at all time points after SAH compared with sham treatment (*P* = 0.0086, *P* < 0.0001, *P* < 0.0001, *P* < 0.0001).

**Figure 4:**
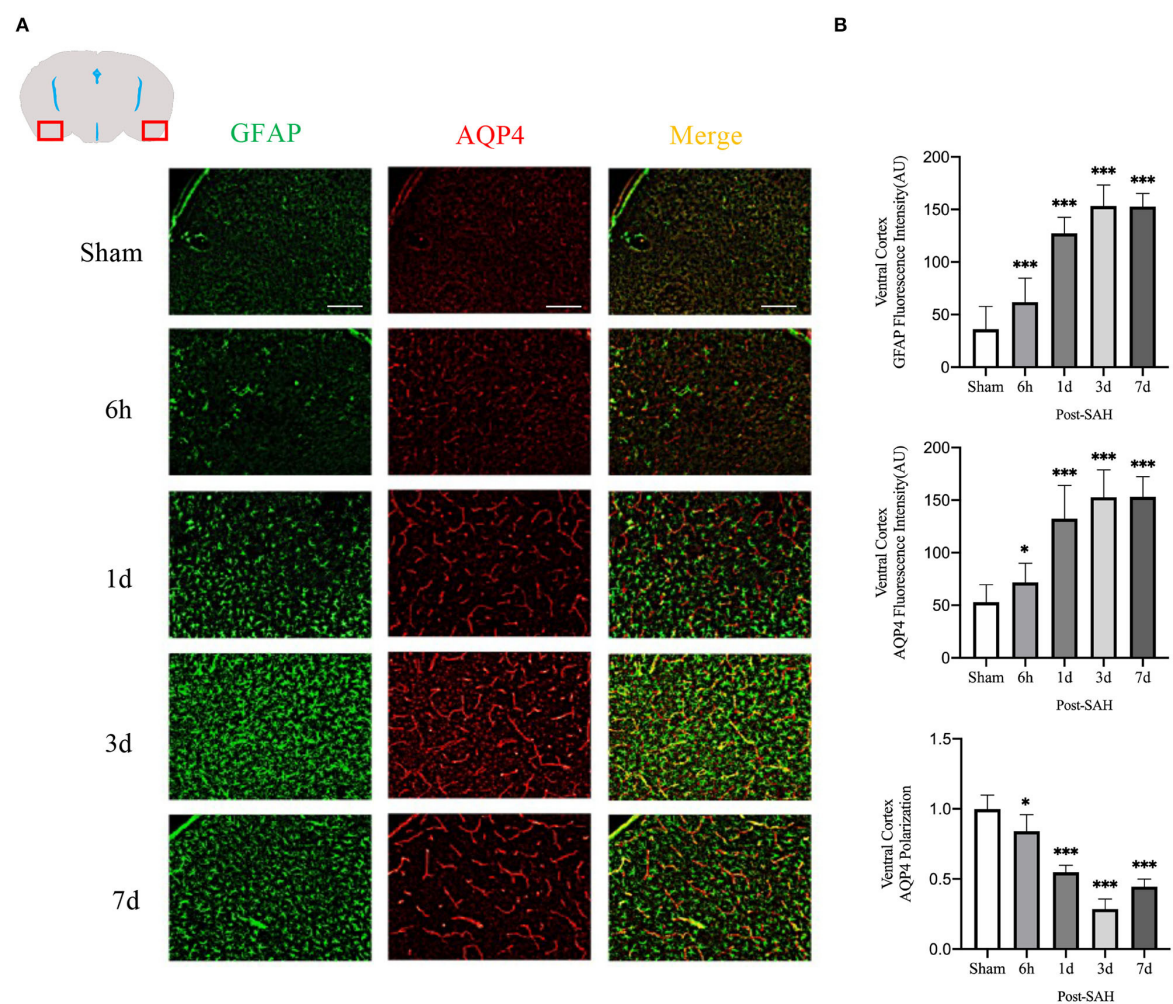
Ventral cortex plots of AQP4 and GFAP fluorescence among different groups: **(A)** Schematic of ventral cortex along with representative immunofluorescence plots; Scale bar, 100 μm; **(B)** Statistical plots of AQP4 mean fluorescence intensity, GFAP mean fluorescence intensity and polarity of AQP4 in the ventral cortex of different groups of mice. Compared with sham group, the mean fluorescence intensity of AQP4 was significantly higher at 6 h, 1d, 3d, and 7d (*P* = 0.0349, *P* < 0.0001, *P* < 0.0001, *P* < 0.0001; *n* = 20–35/group). Compared with sham group, the mean fluorescence intensity of GFAP was significantly higher at 6 h, 1d, 3d, and 7d (*P* < 0.0001, *P* < 0.0001, *P* < 0.0001, *P* < 0.0001; *n* = 20–35/group). Compared with sham group, the polarity of AQP4 was significantly decreased at 6 h, 1d, 3d, and 7d (*P* = 0.0493, *P* < 0.0001, *P* < 0.0001, *P* < 0.0001; *n* = 5/group). *P < 0.05, ***P < 0.001; ns, not statistically significant. One-way ANOVA, Tukey's *post-hoc* test.

The mean fluorescence intensity of AQP4 and GFAP and the polarity of AQP4 were calculated for the dorsal cortex, as shown in Figures 4A,B. Analysis of the mean fluorescence intensity of AQP4 showed that the mean CSF tracer penetration intensity at 6 h, 1, 3, and 7 days after SAH was significantly higher than that of the sham group (*P* = 0.0349, *P* < 0.0001, *P* < 0.0001, *P* < 0.0001). Analysis of the mean fluorescence intensity of GFAP showed that the mean fluorescence intensity at 6 h, 1, 3, and 7 days after SAH was significantly higher than that of the sham group (*P* < 0.0001, *P* < 0.0001, *P* < 0.0001, *P* < 0.0001). Analysis of the polarity of AQP4 revealed that the polarity of AQP4 was significantly decreased at all time points after SAH compared with sham treatment (*P* = 0.0493, *P* < 0.0001, *P* < 0.0001, *P* < 0.0001).

**Figure 5:**
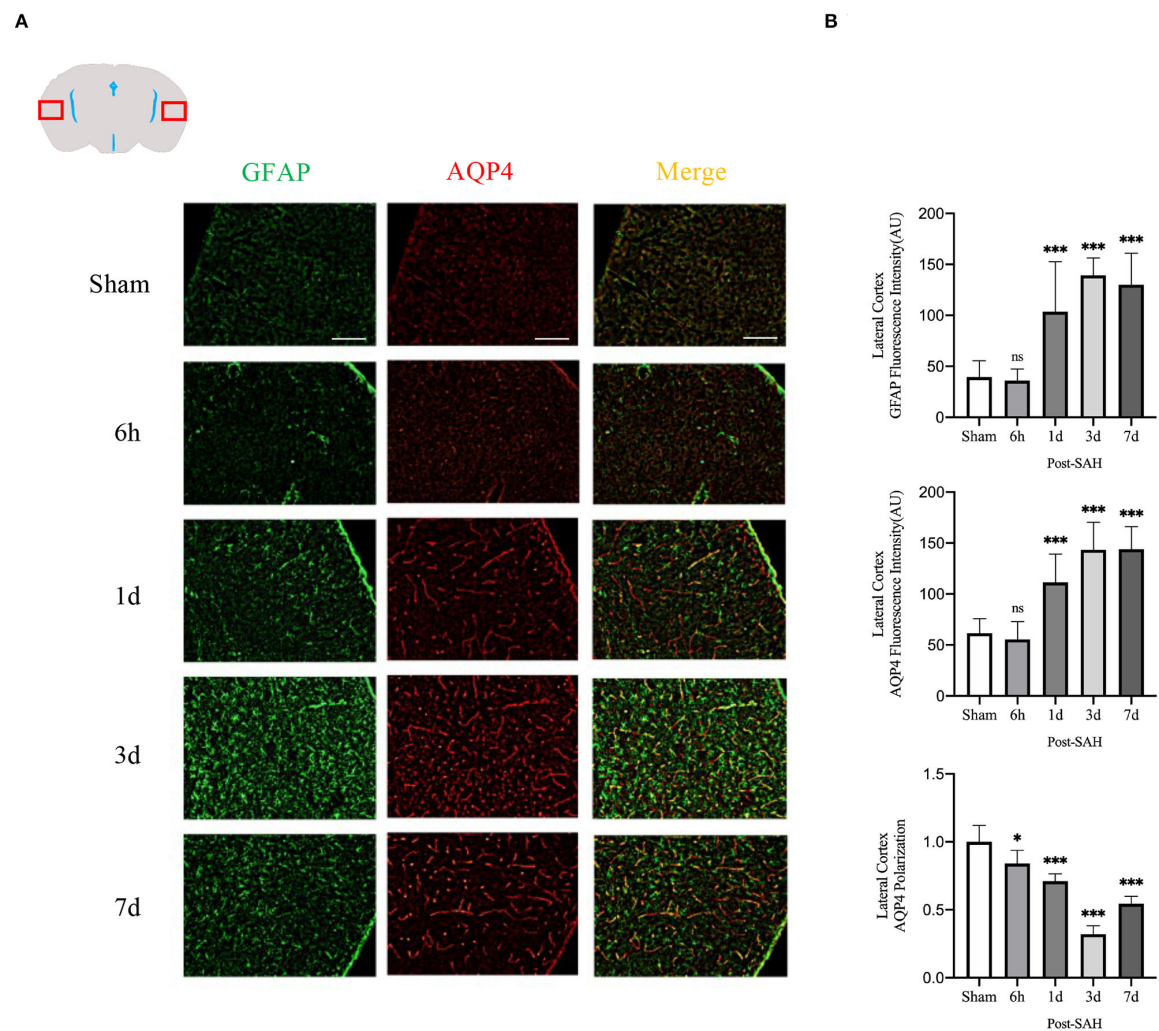
Lateral cortex AQP4 and GFAP fluorescence maps among different groups. **(A)** Schematic of the lateral cortex along with representative immunofluorescence maps. Scale bar, 100 μm; **(B)** Statistical plots of AQP4 mean fluorescence intensity, GFAP mean fluorescence intensity and polarity of AQP4 in the lateral cortex of different groups of mice. Compared with the sham group, the mean fluorescence intensity of AQP4 did not change significantly at 6 h, but increased significantly at 1, 3, and 7 days (*P* = 0.9214, *P* = 0.0007, *P* < 0.0001, *P* < 0.0001; *n* = 16–30/group). Compared with the sham group, the mean fluorescence intensity of GFAP did not change significantly at 6 h but increased significantly at 1, 3, and 7 days (*P* = 0.9960, *P* < 0.0001, *P* < 0.0001, *P* < 0.0001; *n* = 16–30/group). Compared with the sham group, the polarity of AQP4 was significantly decreased at 6 h, 1, 3, and 7 days (*P* = 0.0366, *P* = 0.0001, *P* < 0.0001, *P* < 0.0001; *n* = 5–6/group). *P < 0.05, ***P < 0.001; ns, not statistically significant. One-way ANOVA, Tukey's *post-hoc* test.

The mean fluorescence intensity of AQP4 and GFAP and the polarity of AQP4 were calculated for the lateral cortex, as shown in Figures 5A,B. Analysis of the mean fluorescence intensity of AQP4 showed that there were no significant differences between the 6-h group and the sham group (*P* = 0.9214), but that at 1, 3, and 7 days, it was significantly higher than that of the sham group (*P* = 0.0007, *P* < 0.0001, *P* < 0.0001). Analysis of the mean fluorescence intensity of GFAP showed that the mean fluorescence intensity at 6 h after SAH was not significantly different from that of the sham group (*P* = 0.9960) and that, at 1, 3, and 7 days, it was significantly higher than that of the sham group (*P* < 0.0001, *P* < 0.0001, *P* < 0.0001). Analysis of the polarity of AQP4 revealed that the polarity of AQP4 was significantly decreased at all time points after SAH compared with the sham (*P* = 0.0366, *P* = 0.0001, *P* < 0.0001, *P* < 0.0001).

Thus, although the expression in the dorsal cortex, ventral cortex, and lateral cortex varied, AQP4 expression, GFAP expression, and polarity of AQP4 showed relatively consistent trends. The relative expression of GFAP gradually increased after SAH, peaked at 3 days, and began to decrease at 7 days, associated with increased astrocyte reactivity after inflammation. The relative expression of AQP4 showed a similar change in trend as that of GFAP. The polarity of AQP4 began to decline, reached the lowest value at 3 days after SAH, and then began to recover, but it was still significantly lower than normal.

Subsequently, the protein levels of GFAP and AQP4 among the five groups were validated, as shown in Figure 2B. The relative expression of GFAP in the 6 h, 1, 3, and 7 days groups after SAH was higher than that in the sham group (*P* = 0.0228, *P* = 0.0013, *P* < 0.0001, *P* < 0.0001). However, compared with the sham group, the protein expression of AQP4 in the SAH group at 6 h and 1 day was not significantly increased (*P* = 0.9994, *P* = 0.8447), whereas the relative expression of AQP4 in the 3- and 7-days groups was significantly increased (*P* < 0.0001, *P* < 0.0001). These trends in GFAP and AQP4 were in general agreement with those of the immunofluorescence results.

**Figure 6:**
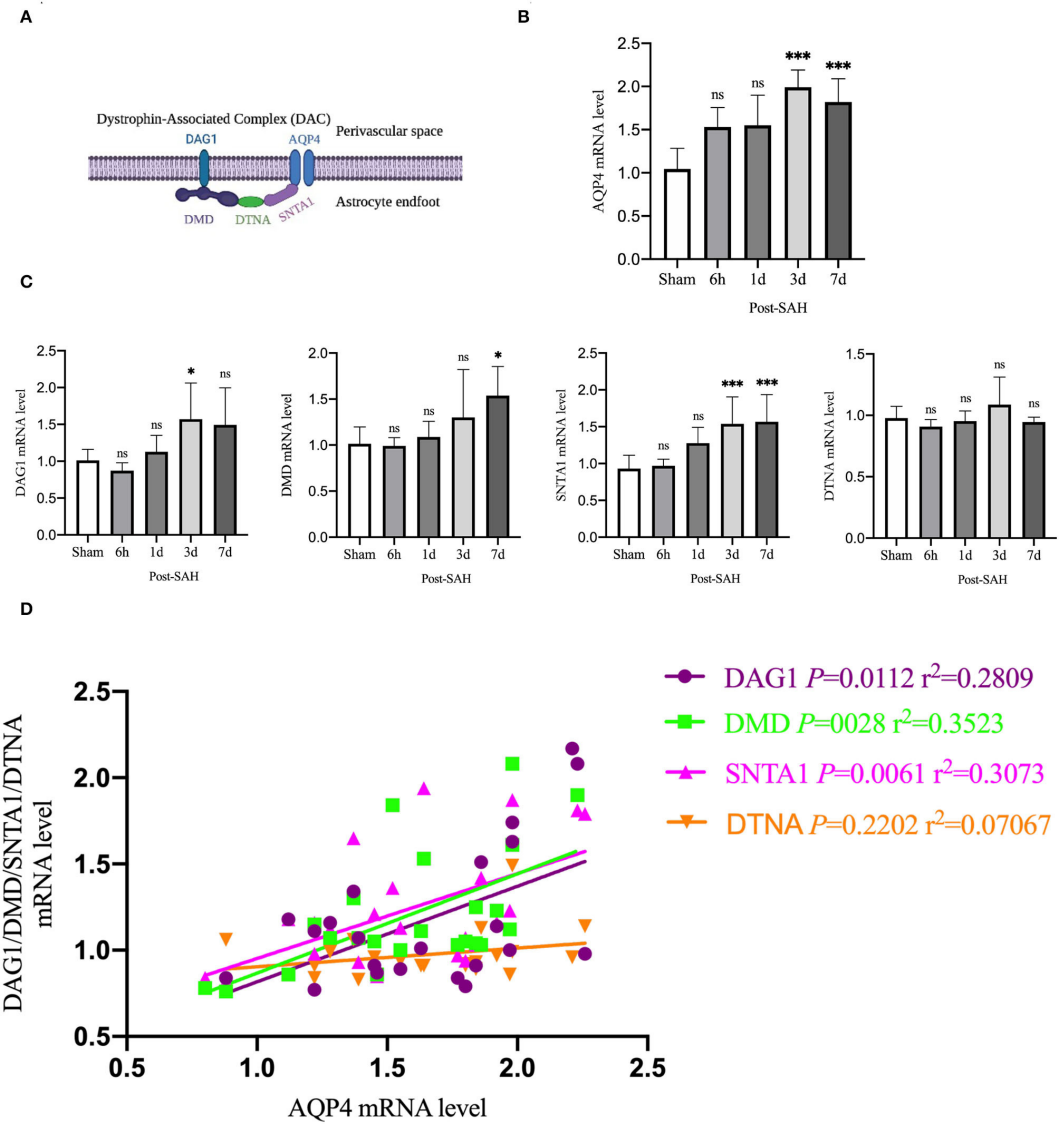
qPCR results for AQP4- and DAC-associated proteins. **(A)** Schematic of DAC and AQP4 ligation. **(B)** qPCR results for AQP4 in the whole cortex 6 h, 1, 3 and 7 days after SAH in Group a vs. sham, *n* = 4–6/group. **(C)** qPCR results of DAC-related proteins in the whole cortex 6 h, 1, 3, and 7 days groups after SAH compared with the sham group. Relative mRNA expression of DMD, *n* = 4–8/group. Relative mRNA expression of DAG1, *n* = 4–7/group. Relative mRNA expression of SNTA1, *n* = 4–8/group. Relative mRNA expression of DTNA, *n* = 4–6/group. **(D)** RNA expression correlation of AQP4 with DMD, DAG1, SNTA1 and DTNA. *P < 0.05, ***P < 0.001; ns, not statistically significant. One-way ANOVA, Tukey's *post-hoc* test.

Aquaporin-4 is a key protein of the GS, so we performed real-time qPCR analysis of the mRNA levels of AQP4 among the groups, as shown in Figure 6B. Compared with the sham group, the mRNA expression of AQP4 in the 6 h and 1 day groups after SAH was not significantly increased (*P* = 0.0557, *P* = 0.0562), whereas it was significantly higher in the 3- and 7-day groups (*P* < 0.0001, *P* = 0.0011). The expression of AQP4 increased immediately and remained at a higher level in the next few days after SAH, corresponding to the increase in the protein level in the western blot analysis, as shown in Figure 2B.

Aquaporin-4 is anchored to the end of perivascular astrocyte foot processes by a dystrophin-associated complex (DAC), which is composed of DMD (dystrophin), DAG1 (dystroglycan), SNTA1 (α-syntrophin), and DTNA (dystrobrevin), as shown in Figure 6A. DAC is necessary for AQP4 to be located at the end of the foot of the astrocyte. Therefore, we evaluated the mRNA expression of DMD, DAG1, SNTA1, and DTNA, as shown in Figure 6C. Compared with the sham group, the mRNA expression of DMD at 6 h, 1, and 3 days after SAH was not significantly different (*P* = 0.9999, *P* = 0.9869, *P* = 0.4517), but that of the 7 days group was significantly higher (*P* = 0.0138). Compared with the sham group, the mRNA expression of DAG1 at 6 h, 1, and 7 days after SAH was not significantly different (*P* = 0.9118, *P* = 0.9518, *P* = 0.1057), but the mRNA expression of the 3 days group was significantly higher (*P* = 0.0439). Compared with the sham group, the mRNA expression of snta1 at 6 h and 1 days after SAH was not significantly different (*P* = 0.9985, *P* = 0.0859), but it was significantly higher on 3 and 7 days (*P* = 0.0033, *P* = 0.0020). Compared with the sham group, the mRNA expression of DTNA at 6 h, 1, 3, and 7 days after SAH in the sham group was not significantly increased (*P* = 0.9206, *P* = 0.9987, *P* = 0.6962, *P* = 0.9964).

To determine whether changes in the mRNA expression of AQP4 were associated with the mRNA expression of DAC after SAH, linear regression analysis was performed on paired data that comprised Figures 6B,C. Linear regression in Figure 6D revealed a significantly positive association among the mRNA expression levels of DMD, DAG1, SNTA1, and AQP4 in the present experiment (*P* = 0.0028, *r*^2^ = 0.3523; *P* = 0.0112, *r*^2^ = 0.2809; *P* = 0.0061, *r*^2^ = 0.3073). The mRNA expression of DTNA was not significantly associated with the mRNA expression of AQP4 (*P* = 0.2202, *r*^2^ = 0.07067). These data indicated that the suppressed expression of DAC could contribute to the loss of polarity of AQP4 after SAH.

**Figure 7:**
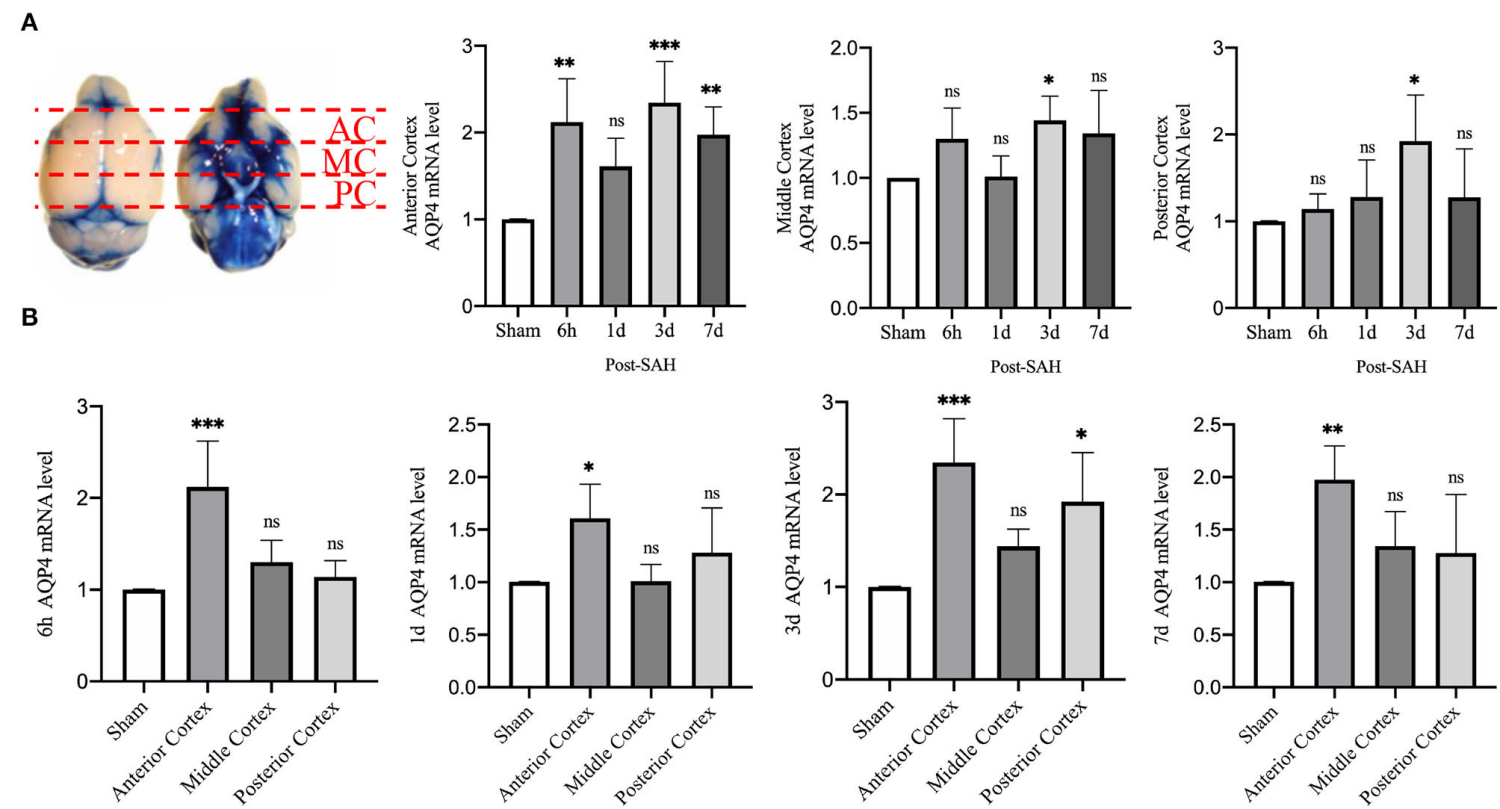
qPCR results for AQP4 at different sites. **(A)** Schematic of the anterior, middle, and posterior cortices; qPCR results for AQP4 in the anterior, middle, and posterior cortices. **(B)** qPCR results of AQP4 in the 6 h, 1, 3, and 7 days groups after SAH; *n* = 4–5/group. *P < 0.05, **P < 0.01, ***P < 0.001; ns, not statistically significant. One-way ANOVA, Tukey's *post-hoc* test.

Then, we explored whether there were differences in the expression of AQP4 mRNA in different regions of the cerebral cortex. We divided the mouse cortex into the anterior cortex, middle cortex, and posterior cortex, and performed real-time qPCR, as shown in Figure 7A.

Statistical analysis of the anterior cortex revealed that the relative mRNA expression of AQP4 at 6 h, 3, and 7 days was higher than that in the sham group (*P* = 0.0025, *P* = 0.0004, *P* = 0.0088), while the relative expression in the 1 day group did not increase significantly (*P* = 0.1606). Statistical analysis in the middle cortex found that the relative mRNA expression of AQP4 was higher in the 3 days group than in the sham group (*P* = 0.0483), whereas no significant difference was observed in the other 3 groups compared with the sham group (*P* = 0.2800, *P* > 0.9999, *P* = 0.1754). Statistical analysis of the posterior cortex revealed that the relative mRNA expression of AQP4 was higher in the 3 days group than in the sham group (*P* = 0.0251), whereas no significant difference was observed in the other three groups compared with the sham group (*P* = 0.9858, *P* = 8461, *P* = 0.8526).

We attempted to explore the relative expression of AQP4 mRNA at different time points, as shown in Figure 7B. The mRNA results from mice at 6 h after SAH showed that compared with the sham group, the mRNA levels of AQP4 in the anterior cortex were significantly higher (*P* = 0.0003), while no significant differences were observed in the middle and posterior cortices (*P* = 0.4738, *P* = 0.8981). The mRNA results of mice from the 1-day group showed that, compared with the sham group, the relative amount of AQP4 mRNA in the anterior cortex was higher (*P* = 0.0304), while no significant difference was observed in the middle and posterior cortices (*P* > 0.9999, *P* = 0.4916). The mRNA results from mice at 3 days showed that compared with the sham group, the relative RNA amounts of AQP4 were higher in the anterior cortex and posterior cortex (*P* = 0.0005, *P* = 0.0119) but not in the middle cortex (*P* = 0.3438). The mRNA results of mice from the 7 days group showed that compared with the sham group, the relative amount of AQP4 mRNA in the anterior cortex was higher (*P* = 0.0071), but not in the middle or posterior cortex (*P* = 0.5441, *P* = 0.6949). This outcome further indicates that the expression of AQP4 in the anterior cortex is significantly higher than that in other parts.

### Early Evaluation of Neurobehavior After SAH

To evaluate the dynamic changes in neurobehavior after SAH, cerebral perfusion, intracranial pressure, brain water content, and neurobehavioral function were tested.

**Figure 8:**
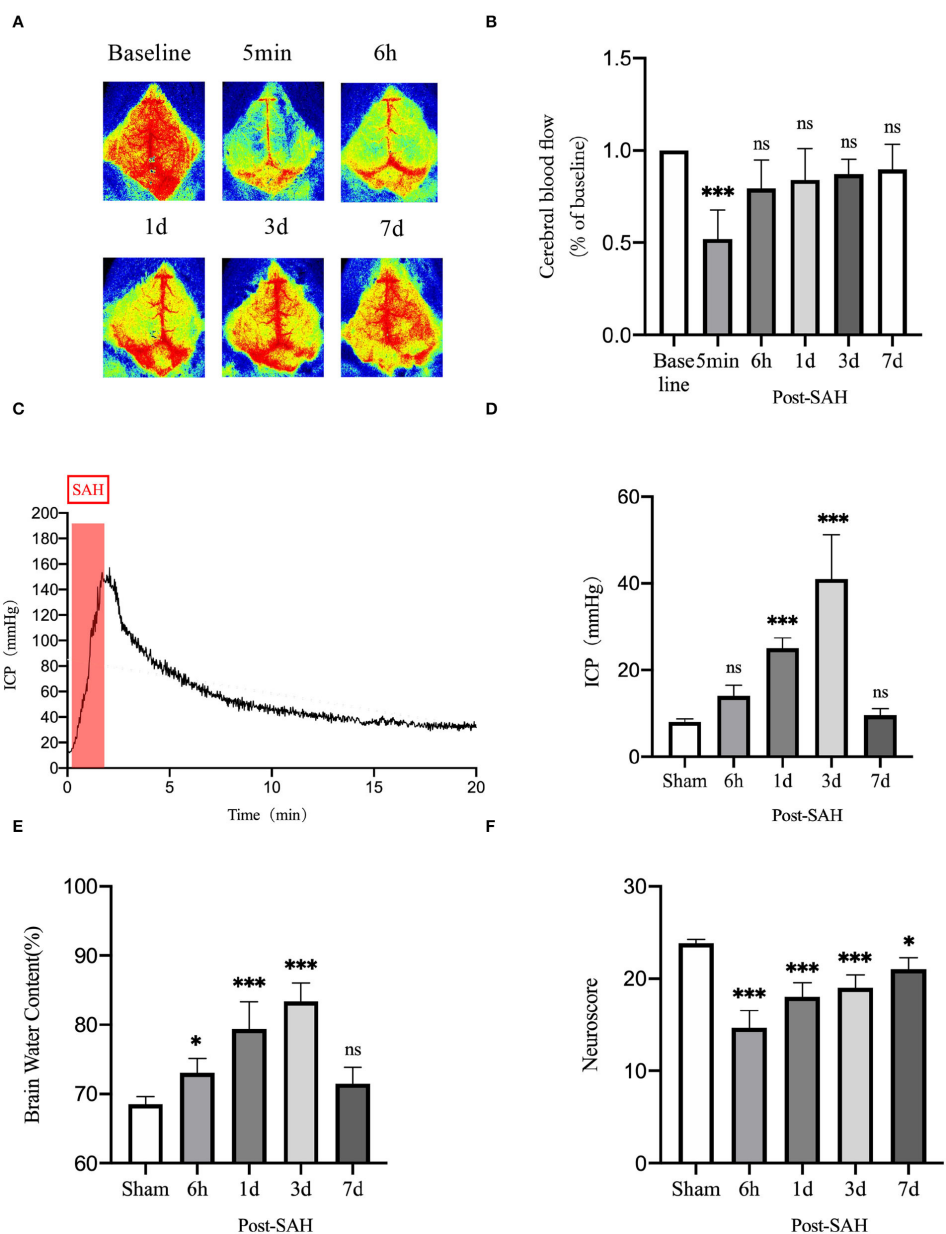
Neurobehavioral function judgment of mice among different groups. **(A)** Schematic of laser speckle in the same mouse at different time points; **(B)** Statistical plots of laser speckle perfusion results in mice, with all results compared to baseline values, *n* = 5/group. **(C)** SAH model mice with real-time intracranial pressure monitoring. **(D)** Statistical plots of intracranial pressure results at different time points in mice after SAH, all compared with the sham group, *n* = 5–6/group. **(E)** Statistical results of mouse brain water content, *n* = 6/group. **(F)** Mouse neurological scoring results, *n* = 6/group; *P < 0.05, ***P < 0.001; ns, not statistically significant. One-way ANOVA, Tukey's *post-hoc* test.

CBF was measured using a laser speckle imager, which was used to continuously observe the cortical blood flow pre-operatively at 5 min, 6 h, 1, 3, and 7 days post-operatively in each mouse. Representative pictures are shown in Figures 8A,B. Cerebral perfusion at 5 min after SAH was significantly lower than the baseline value in each mouse (*P* = 0.0007), whereas it was not significantly different at 6 h, 1, 3, or 7 days after SAH (*P* = 0.1678, *P* = 0.4095, *P* = 0.6384, and *P* = 0.8070). After SAH, the perfusion volume of each mouse decreased to a certain extent relative to the baseline value, especially after the initial stage of SAH. The volume of cerebral perfusion decreased sharply and gradually recovered, but it was not completely normal until 7 days.

Intracranial pressure is one of the GS functional impact factors. Therefore, ICP is one of the concerns for these mice in this experiment. The real-time ICP monitoring curve after SAH is shown in Figure 8C. ICP increased sharply after SAH began to be induced and gradually decreased thereafter. As shown in Figure 8D, compared with the sham group, the 6-h and 7-days groups did not show significantly higher ICP (*P* = 0.3412, *P* = 0.9877), but the 1- and 3-days groups had significantly higher ICP (*P* = 0.0003, *P* < 0.0001). There was a process of acute elevation of ICP in mice after SAH, after which ICP gradually decreased, which should be related to autoregulation in mice, and it returned to a relatively low level at 6 h. However, ICP gradually increased and reached the highest level at 3 days, after which it gradually recovered at 7 days.

The brain water content of the mice is shown in Figure 8E. Compared with the sham group, the brain water content of the 6 h, 1, and 3 days groups increased (*P* = 0.0400, *P* < 0.0001, and *P* < 0.0001), whereas that of 7 days mice did not show an obvious increasing trend (*P* = 0.2984). This unimodal pattern of change was in good agreement with the trend of the intracranial pressure, indicating that the increase in intracranial pressure could be a vasogenic cause of edema, while the increase in intracranial pressure would also decrease the cerebral perfusion volume and cause ischemic hypoxia, further aggravating cerebral edema.

As shown in Figure 8F, the neurological score results showed that the neurological function of mice was significantly worse after SAH, and the decline was obvious in the 6 h, 1, 3, and 7 days groups compared with the sham group (*P* < 0.0001, *P* < 0.0001, *P* < 0.0001, *P* = 0.0128). The reason for the recovery after the first 3 days is not obvious. We speculate that this finding could be related to the progressive increase in ICP and cerebral edema. After 7 days, neurological function recovered obviously.

## Discussion

Subarachnoid hemorrhage is a severe subtype of stroke with high mortality and disability rates worldwide, and it is mainly caused by the rupture of intracranial aneurysms (18, 19). As the disease progresses, there is an increasing burden on society, including the economy. Currently, the developments of microscopic technology, drug support, and medical experience in departments of neurosurgery have played a positive role in the early diagnosis, early treatment, and post-operative care of SAH, but the serious social problems brought by SAH cannot be ignored (20). Studies have shown that the complex physiopathological mechanisms following EBI are essential for the development of prognosis in the course after SAH, rendering the systematic study of the early stage of SAH very necessary (21–23).

The GS has been proved to drain interstitial metabolizing waste, and its function is different under different physiological and pathological conditions (24–26). Due to the previous knowledge of brain tissue as an immune exemption organ, the discovery of this system has enabled scholars to see potential targets for human brain regulation. An increasing number of studies have shown that the function of the GS is disrupted to varying degrees in different diseases, and the resulting adverse outcomes have occurred, e.g., in Alzheimer's disease (AD) and traumatic brain disease (TBI) with long-term disruption of GS function (13, 27, 28). There are certain similarities between our findings and their study, which found that GS function was significantly disrupted early after SAH and that it did not fully recover until day 7 after SAH.

Tau protein is a microtubule-associated protein that stabilizes neuronal microtubules under normal physiological conditions (29, 30). However, under pathological conditions, tau can be phosphorylated to produce abnormal aggregates that are toxic to neurons, often referred to as tau protein disease (31, 32). Studies have shown that, after GS function is impaired, excretion insufficiency can lead to abnormal accumulation of metabolic waste, such as tau protein. This outcome has been verified in cerebral hemorrhage, TBI, AD, and other diseases (33, 34). In this experiment, SAH reduced the GS function of mice. Subsequently, we used western blotting to verify the protein level of phosphorylated tau. The results confirmed our conjecture that reduced GS function after SAH induced a large accumulation of P-tau, which is inevitable for neuronal toxicity. Notably, the excretion of interstitial metabolic waste caused by GS reduction could further damage the function of the GS by damaging neurons, thereby forming a negative cycle.

Numerous studies have shown that AQP/GFAP expression and AQP4 polarization are critical for the function of the GS (5, 24, 33). In our study, cortical sites, including the ventral, dorsal, and bilateral cortices, showed consistent trends. After SAH, there was an increase in AQP4/GFAP expression and a severe and long-term decline in AQP4 polarity. Neuroinflammation and reactive astrogliosis are prominent features of SAH, and reactive astrogliosis is generally associated with the loss of AQP4 expression (14). Current data have already shown that the loss of AQP4 polarity attenuates the ability of interstitial metabolites to be transported, but does the retained metabolic waste interfere with AQP4 polarity by further aggravating neuroinflammation? Studies have shown that the progression of inflammation can interfere with GS function, causing the accumulation of amyloid (5). We speculate that this accumulation might be due to the aggravation of neuroinflammation that caused the proliferation of reactive astrocytes and thus impaired the polarity of AQP4, attenuating GS function.

Our study identified different GS functions at different levels of the brain, mainly reflected by anterior brain tissue tending to have more active GS functions. However, qPCR analysis revealed that AQP4 was more highly expressed in the anterior cortex than in other cortical sites after SAH. This zonal tissue variability in GS function and AQP4 expression might be caused by the unequal distribution of blood after SAH and might also be related to the different physiological functions of the different brain tissue zonations, warranting further attention (35–37). The DAC is critically important for the subcellular localization of AQP4. We found that AQP4 expression was correlated with DAG1, DMD, and SNTA1, which are components of the DAC. Previous studies have shown that AQP4 is directly tethered to snta1 and that snta1 and DAG1 and DMD are linked by DTNA, but no correlation was observed between DTNA and AQP4 in this study. An insufficient amount of DTNA expression was caused after SAH, and the resulting growth rate of the DAC complex with AQP4 could be one of the key factors affecting AQP4 polarization. However, currently, our study cannot directly reveal the relationship between AQP4 polarity and DAC complexes, which deserves further exploration in future studies.

Acute increases in ICP not only disrupt the function of the meningeal lymphatic system in TBI mice and thereby aggravate neuroinflammation and cognitive dysfunction but also impair the function of the GS (38, 39). In our study, a dramatic increase in ICP occurred within a short period after SAH, consistent with the current trend for an increase in ICP after SAH (4, 40, 41). Afterward, ICP was consistently higher than normal and peaked on the third day. Previous studies have confirmed that an increase in ICP is bound to cause a decrease in the volume of intracranial blood perfusion. For the intracranial semiclosed space, cerebral artery pulsation might be affected. The driving force of cerebrovascular fluid and cerebrospinal fluid exchange in cerebrovascular tissue might be among the reasons why ICP affects the function of GS (42).

To the best of our knowledge, the present experiment is the first study to systematically explore the functional changes of the GS in mice at the early stage after SAH. The present study provides a systematic answer to the early function of the GS, as well as AQP4 expression changes after SAH. However, it cannot be denied that the present study has some deficiencies. First, whether the function of the GS could be affected after the accumulation of interstitial metabolic waste products and the extent of the effect were not explored. Second, there is no further discussion of the physiological functions of different brain tissues, GS function, and the expression of AQP4. Finally, it remains to be elucidated as to what extent DAC is associated with AQP4 polarization.

## Conclusions

Our research indicates that the GS function of SAH mice is severely damaged at the early stage, accompanied by the accumulation of tau protein in the interstitium. At the same time, the activation of AQP4 and GFAP and damage to AQP4 polarity were also observed. For a more accurate assessment of the GS, the choice of multiple anatomic levels and different functional sites is necessary. The amount of AQP4 expression varies at different cortical sites, and there is a correlation between AQP4 and the amount of DAC partial protein expression, but the correlation between AQP4 polarity and DAC protein deserves further investigation.

## Data Availability Statement

The raw data supporting the conclusions of this article will be made available by the authors, without undue reservation.

## Ethics Statement

The animal study was reviewed and approved by Tianjin Medical University General Hospital Animal Care and Use Committee.

## Author Contributions

XY, CH, and JL: experimental design. CH and QL: functional measurement of the glymphatic system. JL, HZ, and WW: immunofluorescence and WB of AQP4 and GFAP. CH and BW: SAH mouse modeling. JL and YZ: behavioral experiments in mice and measure the intracranial pressure. XC and WR: statistical analysis. XY, CH, JL, and QL: manuscript writing. All authors: final approval of manuscript. All authors contributed to the article and approved the submitted version.

## Funding

This work was supported by the Natural Science Foundation of Tianjin (No. 20JCZDJC00300) and Tianjin Medical University Clinical Research Program (No. 2018kylc008).

## Conflict of Interest

The authors declare that the research was conducted in the absence of any commercial or financial relationships that could be construed as a potential conflict of interest.

## Publisher's Note

All claims expressed in this article are solely those of the authors and do not necessarily represent those of their affiliated organizations, or those of the publisher, the editors and the reviewers. Any product that may be evaluated in this article, or claim that may be made by its manufacturer, is not guaranteed or endorsed by the publisher.

## Abbreviations

SAH: subarachnoid hemorrhage
GS: glymphatic system
AQP4: aquaporin-4
GFAP: glial fibrillary acidic protein
DAC: dystrophin-associated complex
DMD: dystrophin
DAG1: dystroglycan
SNTA1: α-syntrophin
DTNA: dystrobrevin
CSF: cerebrospinal fluid
EBI: early brain injury
ICP: intracranial pressure
AD: Alzheimer's disease
TBI: traumatic brain disease.
