# Supplementary material for: Dynamic Evolution of the Glymphatic System at the Early Stages of Subarachnoid Hemorrhage
Source: Front Neurol. 2022 Jul 1;13:924080. doi: 10.3389/fneur.2022.924080 (PMC9283644; doi:10.3389/fneur.2022.924080)
Supplement: Supplementary file 1 [file Data_Sheet_1.pdf]

Supplementary Materials

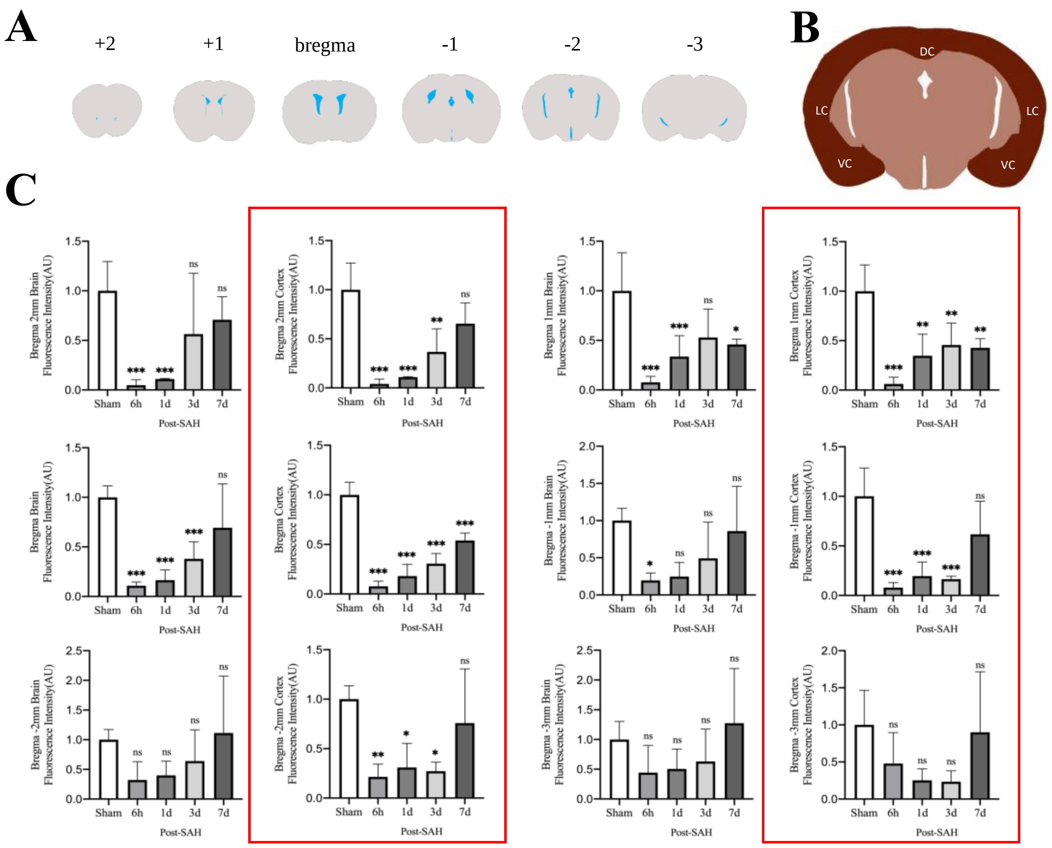

Supplementary Figure 1. GS CSF tracer penetration images and statistical results.

(A) Schematic representation of different layers of fluorescent tracers;

(B) Schematic of cortex; DC: dorsal cortex, VC: ventral cortex, LC: lateral cortex;

(C) Full-section CSF tracer penetration intensity and cortical CSF tracer penetration intensity (red box) statistics at the following locations: bregma +2 mm, bregma +1 mm, bregma, bregma -1 mm, bregma -2 mm, bregma -3 mm. n = 4-5/group, $*P < 0.05$, $**P < 0.01$, $***P<0.001$; ns = not statistically significant. One-way ANOVA, Tukey's post-hoc test.

GS dysfunction appears in early stages after SAH

We assessed GS function in sham, 6 h, 1 d, 3 d, and 7 d mice using cisterna magna injection of a fluorescent CSF tracer. For a more accurate analysis of GS function, we analyzed the whole-slice CSF tracer penetration and cortical CSF tracer penetration intensity of 6 different anatomical layers, including bregma +2 mm, bregma +1 mm, bregma, bregma -1 mm, bregma -2 mm and bregma -3 mm.

The whole-slice CSF tracer penetration intensity at bregma +2 mm of each mouse brain was evaluated using Fiji, and the 6 h, 1 d, 3 d, and 7 d groups after SAH were compared with the sham group. As shown in Supplement Figure 1C, the CSF tracer penetration intensity at 6 h and 1 d after SAH was significantly weaker than that in the sham group(P = 0.0027, P = 0.0076), but no significant difference was observed between the 3 D and 7 d groups after SAH compared with the sham group(P = 0.3227, P = 0.6834). The whole-slice CSF tracer penetration intensity at bregma +1 mm of each mouse brain was evaluated using Fiji, and the 6 h, 1 d, 3 d, and 7 d groups after SAH were compared with the sham group. As shown in Supplement Figure 1C, the CSF tracer penetration intensity at 6 h, 1 d and 7 d after SAH was significantly weaker than that in the sham group (P=0.0002, P = 0.0066, P = 0.0299), but no significant difference was observed between the 3 d group and the sham group (P = 0.0685).

The whole-slice CSF tracer penetration intensity at the bregma of each mouse brain was evaluated using Fiji, and the 6 h, 1 d, 3 d, and 7 d groups after SAH were compared with the sham group. As shown in Supplement Figure 1C, the CSF tracer penetration intensity at 6 h, 1 d and 3 d after SAH was significantly weaker than that in the sham group (P=0.0001, P=0.0004, P=0.0071), but no significant difference was observed between the 7 d group and the sham group (P=0.3095).

The whole-slice CSF tracer penetration intensity at bregma -1 mm of each mouse brain was evaluated using Fiji, and the 6 h, 1 d, 3 d, and 7 d groups after SAH were compared with the sham group. As shown in Supplement Figure 1C, the CSF tracer penetration intensity at 6 h after SAH was significantly weaker than that in the sham group (P=0.0278), but no significant difference was observed among the 1 d, 3 d and 7 d groups compared with the sham group (P=0.0576,P=0.3025,P=0.9789).

The whole-slice CSF tracer penetration intensity at bregma -2 mm of each mouse brain was evaluated using Fiji, and the 6 h, 1 d, 3 d, and 7 d groups after SAH were compared with the sham group. As shown in Supplement Figure 1C, no significant differences were observed among the 6 h, 1 d, 3 d and 7 d groups compared with the sham group (P=0.3248,P=0.4890,P=0.8565,P=0.9977).

The whole-slice CSF tracer penetration intensity at bregma -3 mm of each mouse brain was evaluated using Fiji, and the 6 h, 1 d, 3 d, and 7 d groups after SAH were compared with the sham group. As shown in Supplement Figure 1C, no significant differences were observed among the 6 h, 1 d, 3 d and 7 d groups compared with the sham group (P=0.5740,P=0.7137,P=0.8740,P=0.9528).

Current GS research is more likely to evaluate the cortex. We further analyzed the CSF tracer penetration intensity of the GS in different anatomic layers in mice, and the selected cortical schematic is shown in Supplement Figure 1C.

The cortical CSF tracer penetration intensity at bregma +2 mm of each mouse brain was evaluated using Fiji, and the 6 h, 1 d, 3 d, and 7 d groups after SAH were compared with the sham group. As shown in the red box in Supplement Figure 1C, the CSF tracer penetration intensity at 6 h, 1 d and 3 d after SAH was significantly weaker than that in the sham group (P<0.0001, P<0.0001, P=0.0012), but no significant difference was observed between the 7 d groups after SAH compared with the sham group (P=0.1010).
The cortical CSF tracer penetration intensity at bregma +1 mm of each mouse brain was evaluated using Fiji, and the 6 h, 1 d, 3 d, and 7 d groups after SAH were compared with the sham group. As shown in the red box in Supplement Figure 1C, the CSF tracer penetration intensity at 6 h, 1 d, 3 d and 7 d after SAH was significantly weaker than that in the sham group (P<0.0001, P=0.0011, P=0.0056, P=0.0036).

The cortical CSF tracer penetration intensity at the bregma of each mouse brain was evaluated using Fiji, and the 6 h, 1 d, 3 d, and 7 d groups after SAH were compared with the sham group. As shown in the red box in Supplement Figure 1C, the CSF tracer penetration intensity at 6 h, 1 d, 3 d and 7 d after SAH was significantly weaker than that in the sham group (P<0.0001, P<0.0001, P<0.0001, P<0.0001).

The cortical CSF tracer penetration intensity at bregma -1 mm of each mouse brain was evaluated using Fiji, and the 6 h, 1 d, 3 d, and 7 d groups after SAH were compared with the sham group. As shown in the red box in Supplement Figure 1C, the CSF tracer penetration intensity at 6 h, 1 d and 3 d after SAH was significantly weaker than that in the sham group ($P<0.0001$, $P=0.0003$, $P=0.0002$), but no significant difference was observed between the 7 d groups after SAH compared with the sham group ($P=0.1008$). The cortical CSF tracer penetration intensity at bregma -2 mm of each mouse brain was evaluated using Fiji, and the 6 h, 1 d, 3 d, and 7 d groups after SAH were compared with the sham group. As shown in the red box in Supplement Figure 1C, the CSF tracer penetration intensity at 6 h, 1 d and 3 d after SAH was significantly weaker than that in the sham group ($P=0.0049$, $P=0.0199$, $P=0.0138$), but no significant difference was observed between the 7 d groups after SAH compared with the sham group ($P=0.7297$). The cortical CSF tracer penetration intensity at bregma -3 mm of each mouse brain was evaluated using Fiji, and the 6 h, 1 d, 3 d, and 7 d groups after SAH were compared with the sham group. As shown in the red box in Supplement Figure 1C, no significant difference was observed among the 6 h, 1 d, 3 d and 7 d groups after SAH compared with the sham group ($P=0.4800$, $P=0.2044$, $P=0.1880$, $P=0.9981$).
